# Modeling airport congestion contagion by heterogeneous SIS epidemic spreading on airline networks

**DOI:** 10.1371/journal.pone.0245043

**Published:** 2021-01-22

**Authors:** Alberto Ceria, Klemens Köstler, Rommy Gobardhan, Huijuan Wang

**Affiliations:** 1 Faculty of Electrical Engineering, Mathematics and Computer Science, Delft University of Technology, Delft, The Netherlands; 2 Faculty of Aerospace Engineering, Delft University of Technology, Delft, The Netherlands; Unviersity of Burgundy, FRANCE

## Abstract

In this work, we explore the possibility of using a heterogeneous Susceptible- Infected-Susceptible SIS spreading process on an airline network to model airport congestion contagion with the objective to reproduce airport vulnerability. We derive the vulnerability of each airport from the US Airport Network data as the congestion probability of each airport. In order to capture diverse flight features between airports, e.g. frequency and duration, we construct three types of airline networks. The infection rate of each link in the SIS spreading process is proportional to its corresponding weight in the underlying airline network constructed. The recovery rate of each node is also heterogeneous, dependent on its node strength in the underlying airline network, which is the total weight of the links incident to the node. Such heterogeneous recovery rate is motivated by the fact that large airports may recover fast from congestion due to their well-equipped infrastructures. The nodal infection probability in the meta-stable state is used as a prediction of the vulnerability of the corresponding airport. We illustrate that our model could reproduce the distribution of nodal vulnerability and rank the airports in vulnerability evidently better than the SIS model whose recovery rate is homogeneous. The vulnerability is the largest at airports whose strength in the airline network is neither too large nor too small. This phenomenon can be captured by our heterogeneous model, but not the homogeneous model where a node with a larger strength has a higher infection probability. This explains partially the out-performance of the heterogeneous model. This proposed congestion contagion model may shed lights on the development of strategies to identify vulnerable airports and to mitigate global congestion by e.g. congestion reduction at selected airports.

## Introduction

Networks, ranging from social, transportation to physical contact networks, support the diffusion of information, transportation of goods and spreading of epidemics. Therefore, networks and processes that unfold on them have been investigated in a wide range of fields such as mathematics, engineering and social sciences [1–5]. The Susceptible-Infected-Susceptible (SIS) epidemic spreading process is one of the most studied dynamic processes on networks [6–14]. The classic homogeneous SIS spreading process has been defined as follows. At any time *t*, a node is either susceptible *S* or infected *I*. A susceptible node can be infected by each of its infected neighbors with an infection rate *β*. Each infected node recovers to be susceptible again with a recovery rate *δ*. Both the infection and recovery processes are independent Poisson processes. For a given network upon which the SIS process is deployed, a critical epidemic threshold *τ*_*c*_ exists. When the effective spreading rate *τ* = (*β*/*δ*) > *τ*_*c*_, a non-zero fraction of infected nodes persists in the meta-stable state. When *τ* < *τ*_*c*_, the epidemic dies out. The vulnerability of a network to an epidemic is estimated by the prevalence, defined as the average fraction of infected nodes in the meta-stable state. The infection probability *v*_*i*∞_ of a node *i* indicates its vulnerability to the epidemic. Recent studies have focused on the influence of the underlying network topology and heterogeneous infection/recovery rates on the epidemic threshold, the prevalence [15, 16] and nodal infection probabilities [17]. Epidemic spreading processes have been developed to model e.g. the propagation of epidemic, information, failures and computer worms.

A fundamental question is to what extent an abstract process like the epidemic spreading process could model a generic complex system, i.e. reproduce the key properties of the system. This question is motivated at least from the following perspective. The operating mechanisms of many complex systems like social systems and the brain are far from well understood. A model that could well reproduce the key properties of a complex system may unravel the possible operating mechanism. The operating mechanisms of many complex systems are possibly known, however, too complex to derive optimization/control solutions. In this case, an abstract model that well captures the key features of the system may possibly facilitate the development of optimization solutions.

For airline transportation networks, initial effort has been devoted to the analysis of their topologies, demonstrating properties such as the small-world and scale-free degree distribution [18, 19]. Topological properties of subsets of a network based on geography and airlines/alliances have also been explored [20, 21]. Recent investigations have focused on network resilience and vulnerability regarding random failures [22, 23]. The performance or state of an airport (e.g. congested or not and the average delay per hour) is not independent of the states of other airports. The delay propagation between airports has been studied via e.g. the correlation or causality measures between the time series (average delay per hour) of airports [24–28]. One of the main reasons why delay propagates is that each aircraft has a flight sequence where it travels between possibly multiple airports a day. The congestion at an airport can be introduced by local factors such as the slow boarding of passengers, the mechanical issues of an aircraft at the airport. Beyond, delayed flights that depart from a congested airport could cause an overcharge at the arrival airports. The Air-Traffic Flow Management systems use strategies such as ground holding (intentionally delaying an aircraft’s takeoff) and re-routing to reduce overload [29]. The weather condition could lead to the congestion of several nearby airports, which may further cascade to more airports due the rescheduling or re-routing of aircraft. These perspectives imply the possible contagion of congestion between airports. Airline congestion has been studied via network dynamics like queuing models [30]. Epidemic spreading process has been recently used to model the spreading of traffic jams in urban networks, assuming both homogeneous infection and recovery rate and homogeneous mixing approximation in network topology [31]. The possibility of modeling congestion contagion on an airline network using epidemic spreading process has been barely explored, not to mention how to develop a full-fledged heterogeneous spreading model.

In this work, we explore the possibility and limits of modelling airport congestion contagion by a heterogeneous SIS spreading process on an airline network in reproducing or predicting airport vulnerability. We consider the US Airport Network data [32]. The airport vulnerability is defined as the ratio of the duration of traffic congestion over the total operation time and derived from data. We construct three types of airport networks to capture diverse features such as the frequency and duration of flights. In the heterogeneous SIS model that we proposed, the infection rate of a link is proportional to the weight of the link, as defined in each of the three airline networks. Moreover, the recovery rate of a node is also heterogeneous, dependent on the strength of the node in the underlying network. We use the nodal infection probability in the meta-stable state as an estimation of the corresponding airport’s vulnerability, which will be further compared with the airport vulnerability derived from the US Airport dataset to evaluate our model. Specifically, our model is evaluated according to its capability to reproduce the distribution of the vulnerability of a node and the ranking of nodes in vulnerability. The modeling of airport congestion contagion by the SIS process, where the infection rate of a link is proportional to the weight of the link and the recovery rate is homogeneous, has been explored in [33]. That SIS process is a special case of our heterogeneous model and is called the homogeneous SIS model in this paper to emphasize its homogeneous recovery rate. We illustrate that the heterogeneous SIS model evidently outperforms the homogeneous model according both aforementioned evaluation perspectives. Our further exploration of the infection probability in relation to the node strength of an airport explains the better performance of the heterogeneous model in reproducing the ranking of nodes in vulnerabilities.

We propose and illustrate the basic method to model a complex system by an epidemic spreading process, via the airline system. The relatively good performance of the model does not imply that the derived model is the precise mechanism of congestion contagion. Further verification of the contagion mechanism is needed, e.g. regarding whether nodes with a large strength recover faster. The derived model may inspire the development of strategies to identify vulnerable airports and to mitigate global congestion by e.g. reducing congestion at selected airports.

The content of this paper is arranged as follows. Firstly we define, derive and characterize the airport vulnerability derived from data. Furthermore we introduce the heterogeneous SIS spreading model and network construction. Afterwards, the methods to evaluate the model are presented. In results, we compare the performance of our model with the homogeneous model. The final section summarizes our key findings and discusses possible future work.

## Materials and methods

### Traffic vulnerability of an airport

Firstly, we describe the US Airport Network data. Airport vulnerability and its distribution are further defined and derived respectively. Airport vulnerability obtained from data will be adopted as a benchmark to evaluate the performance of our model.

#### Data

We obtain the U.S. airport dataset from the Bureau of Transportation Statistics (BTS). This data set includes detailed information about the U.S. flight schedules since 1987 [32]. The computer reservation system (CRS) further distinguishes flight schedules as the planned schedule under optimal operation conditions, and the actual schedule. In order to demonstrate our modelling approach, we use the data spanning the high season period from July 1st 2018 to July 14th 2018, since flight schedule and rotations periodically repeat. In total *N* = 349 airports and *E* = 645299 flights have been considered. This data set contains as well extra information for each flight e.g. Tail-number, Origin and Destination, Date, the actual and scheduled Departure/Arrival Times.

#### Definition and statistical properties

The vulnerability of an airport is defined as its duration of traffic congestion over its total operation time, which is its probability of being congested. Per hour, an airport’s declared capacity corresponds approximately to the number of movements (the total number of departure and arrival flights) planned for that hour, such that a reasonable level of service (LOS) can be ensured. Delay is the principal indicator of LOS. Usually the declared capacity of an airport is up to 85–95% of its maximum throughput capacity, which is the maximal number of movements per hour that the airport’s runway system allows according to air traffic management rules and assuming continuous aircraft demands. An airport is considered congested if its actual number of movements per hour during operation is greater than its declared capacity (the planned number of movements) divided by a parameter *α*, where 0.85 ≤ *α* ≤ 1. We consider *α* = 0.9 as an example to illustrate our methods. The state of each airport *i* at each hour *t* is derived from U.S. airport dataset as follows: the airport is congested (*X*_*i*_(*t*) = 1) if the actual number of movements is larger than the number of movement planned at time *t* divided by 0.9. If this condition is not satisfied, the airport is not congested (*X*_*i*_(*t*) = 0). Airport *i*’s vulnerability $\phi_{i}=\frac{1}{m}\sum_{t=1}^{m}X_{i}\left(t\right)$ is the fraction of time that airport *i* is congested. We considered all hours in the previously specified two week’s interval (excluding hours between 0 and 6 of each day due to their low number of movements). The hours considered are indexed as [1, 2, …, *m*], where *m* = 18 ⋅ 14 = 252.

In this work, we confine ourselves to this limited definition of airport vulnerability to start and to illustrate our method. The definition could be further generalized to capture the level of congestion per hour. The declared capacity can also also be better estimated based on airport characteristics (e.g. active runways, taxiways, etc.) and weather conditions, beyond flight schedule.

**Fig 1:**
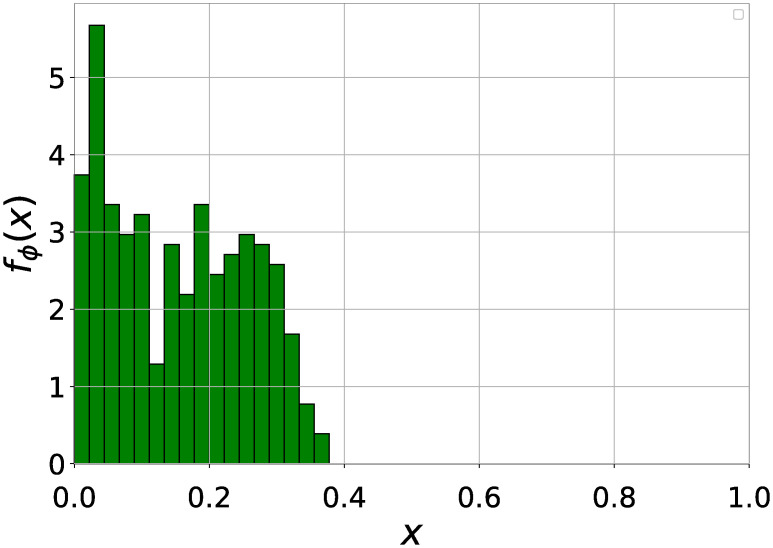
Probability density function *f*_*ϕ*_(*x*) of the vulnerability *ϕ* of an airport. The average vulnerability is *E*[*ϕ*] = 0.15 and the variance is *Var*[*ϕ*] = 0.01. In total 45 bins are split within the interval [0, 1] with the same bin size. The probability density *f*_*ϕ*_(*x*) at a given bin *x* eqals the percentage of the airports whose vulnerability falls within the bin normalized by the bin size 1/45.

Fig 1 shows the distribution of airport vulnerability, whose average is 0.15 and variance is 0.01. The vulnerabilities of all the airports are within the range [0, 0.4].

### Heterogeneous SIS spreading model on airline networks

We model the contagion of airport congestion as a heterogeneous SIS spreading process on an airline network. Firstly we introduce how to construct the three types of airline networks. Secondly, we propose the heterogeneous SIS spreading model. The last subsection illustrates the individual-based mean-field approximation to compute nodal infection probabilities in the meta-stable state, given the underlying network and the model parameters.

#### Network construction and properties

We derive three types of undirected networks from the U.S. Airport Network data over the two weeks’ period in order to capture various flight properties. This is motivated by the fact that the SIS spreading process unfolds differently on different underlying networks. Network *G*_1_ is unweighted: two nodes (airports) are connected if at least one direct flight exists in between. Each existing link has a weight *w*_*ij*_ = 1. Network *G*_2_ and *G*_3_ are both weighted and have the same network topology as network *G*_1_. It is assumed that the infection rate along a link is proportional to the link’s weight. In *G*_2_, the link which connects node *i* and *j* has weight $w_{ij}^{*}=F_{ij}+F_{ji}$, which is the sum of the total number *F*_*ij*_ of flights from *i* to *j* and the number *F*_*ji*_ of flights from *j* to *i* in the two weeks’ period. We motivate this weight definition by the assumption that frequent flights between two airports correspond to a high chance that congestion spreads from one airport to the other. Furthermore, congestion propagation may be affected also by the duration of flights between airports. An airplane that has departed with a delay in time, in fact, can adapt its speed to respect its scheduled arrival time at the destination airport. In order to capture these effects we introduce Network *G*_3_. This network is defined by assigning to each link (*i*, *j*) the weight $w_{ij}^{*}=\frac{1}{E[T_{ij}]}$, which is the inverse of the average flight time between airport *i* and *j*. We adopt the convention that the flight time between airports not connected by any direct flights is infinite: this ensures that the weight of non-existing links is always null. A smaller average flight time may result in a higher chance that flights delayed at the departure airport would affect the arrival time at the destination airport. This situation may be less likely in the case of a larger average flight time, when there is more room for airplanes to re-optimize the flight velocity.

Finally, the weights in Networks *G*_2_ and *G*_3_ are respectively normalized as

$$\begin{array}{c} w_{ij}=(\frac{w_{ij}^{*}}{\max_{k,l}w_{k,l}^{*}}). \end{array}$$

The normalization by the maximum link weight $\max_{k,l}w_{k,l}^{*}$ in each network leads to the normalized link weights within the range (0, 1]. Since there is no self-loop, *w*_*ii*_ = 0 ∀*i*.

**Fig 2:**
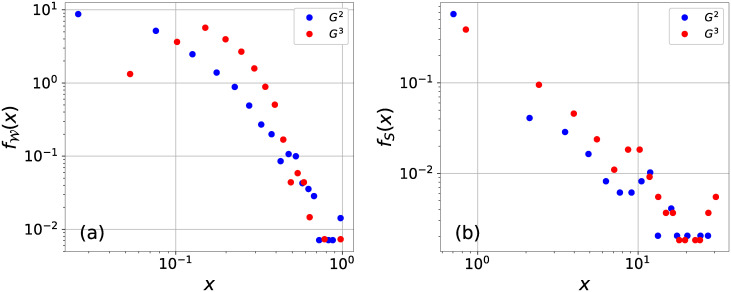
The probability density functions $f_{W}\left(x\right)$ of the weight W of a link (a) and *f*_*S*_(*x*) of the strength *S* of a node (b) in network *G*_2_ (blue points) and *G*_3_ (red points). Horizontal and vertical axes are presented in logarithmic scale. The horizontal axis is split into 20 bins, each with the same bin size in the linear scale. The probability density $f_{W}\left(x\right)$ (*f*_*S*_(*x*)) at a given bin *x* is equal to the fraction of the links (nodes) whose weight (strength) falls within the bin normalized by the bin size. Both link weight and node strength have, respectively, a higher average in *G*_3_ and higher coefficient of variation (the ratio of standard deviation over the average) in *G*_2_.

Heterogeneous infection rate and recovery rate (link weight) have been shown to influence the nodal infection probabilities [11, 17]. Since the infection rate of a link and the recovery rate will later be defined as a function of the link weight and node strength of a node respectively, we examine the distribution of the link weight and node strength (the total weight of the links incident to a node) in Fig 2. Network *G*_2_ and *G*_3_ manifest different link weight and node strength distributions, which motivate again the consideration of the three types of networks that capture different features of the airline system.

We explore relation between the strength of a node and other centrality metrics that describe varies topological properties of a node via the linear correlation coefficient. The following centrality metrics have been considered:

- *Clustering Coefficient*. In an unweighted network, the clustering coefficient is the probability that two random neighbors of a node are connected. In a weighted network, a generalized definition for clustering coefficient has been introduced by [34]. The intensity of a triangle among node *i*, *j* and *k* is defined as $\sqrt[{3}]{w_{ij}w_{jk}w_{ki}}$. The clustering coefficient of a node *i* is then defined as the sum of the intensities of the triangles that *i* resides in, normalized by the maximum possible number of triangles that *i* could reside in, i.e. $\frac{1}{2}d_{i}\left(d_{i}-1\right)$, where *d*_*i*_ is the degree of node *i*.
- *Betweenness Centality*. The betweenness centrality of a node is the fraction of the shortest paths between all possible node pairs that pass through the node. To compute the shortest path between a node pair, we define the distance of each link in the underlying network as the reciprocal of its link weight [35].
- *Closeness Centrality*. The closeness centrality is the average hopcount of a node to any other node. The hopcount between two nodes is the number of links of the shortest path, which is computed as described in betweenness.
- *Principal Eigenvector Component* The principal eigenvector component of a node is its corresponding component in the principal eigenvector of the weighted adjacency matrix. The principal eigenvector is the one corresponding to the largest eigenvalue.

The linear correlation coefficient between node strength and each of centrality metric in the three networks constructed are shown in Table 1.

**Table 1:** The linear correlation coefficient of node strength with clustering coefficient, betweenness, closeness and eigenvector centrality respectively in network *G*_1_, *G*_2_ and *G*_3_.

Network	Clustering	Betweenness	Closeness	Eigenvector
*G*_1_	-0.09	0.80	0.81	0.95
*G*_2_	0.00	0.81	0.54	0.98
*G*_3_	-0.17	0.81	0.44	0.92

Node strength is strongly correlated with all the centrality metrics that describe a given importance of node in the whole network except for the clustering coefficient, a nodal property derived from local network connections. Hence, node strength that will be used to define the nodal recovery rate in the epidemic spreading model, captures as well nodal properties like betweenness, closeness and principal eigenvector component.

**Fig 3:**
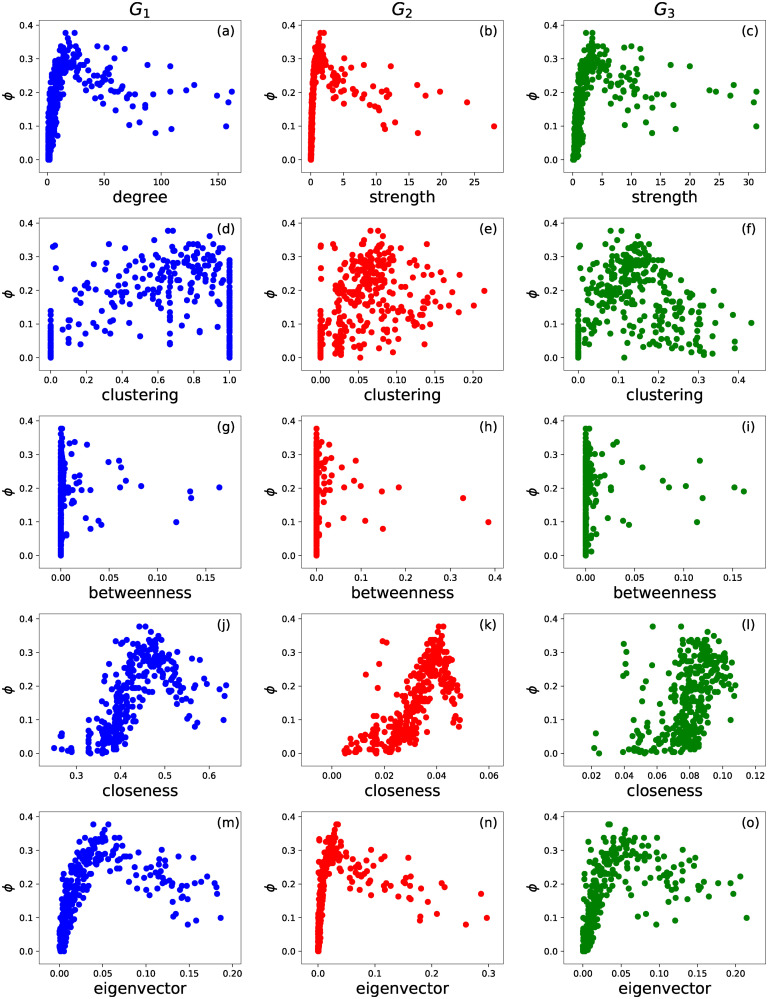
Airport vulnerability versus a nodal centrality metric. The scatter plot of airport vulnerability *ϕ* versus node strength (a,b,c), clustering coefficient (d,e,f), betweenness (g,h,i), closeness (j,k,l) and eigenvector (m,n,o) centrality in network *G*_1_ (first column, blue color), *G*_2_ (second column, red color) and *G*_3_ respectively.

Furthermore, we study the relation between the vulnerability *ϕ* of an airport and a given centrality metric of the corresponding node in each of the three underlying networks. This helps us to evaluate the possibility of using a nodal centrality measure to estimate nodal vulnerability. In the scatter plot in Fig 3, we do not observe any monotonic trend between the vulnerability *ϕ* of an airport and the centrality metric of the corresponding node. This implies that centrality metrics can not be used as a good estimation of airport vulnerability. Our previous work [33] illustrated as well the worse performance of vulnerability prediction via centrality metrics than that via the homogeneous SIS model. Hence, we will compare performance of the heterogeneous SIS model with that of the homogeneous SIS model but not of the centrality metrics.

**Fig 4:**
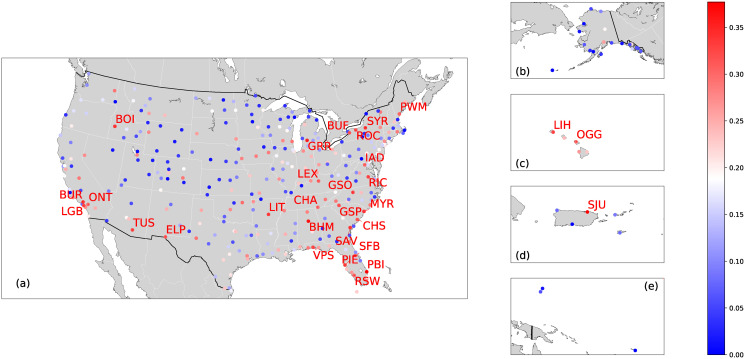
Geographic location and vulnerability of U.S. airports. The geographic location and vulnerability of an airport in U.S. mainland (a), Alaska (b), Hawaii Islands (c), Puerto Rico (d), American Samoa and Guam (e). The nodes/airports are color-coded according to their airport vulnerability *ϕ*. We show the names of the top 30 most vulnerable airports.

The networks we constructed have not taken the geographical locations of the airports explicitly into account. One may wonder whether the vulnerability of an airport may strongly correlate with its location, thus can be possibly estimated by its location. Fig 4 shows that vulnerable airports are scattered in location and no evident relation between vulnerability and location.

#### The heterogeneous SIS model

We model the airport congestion dynamics as a heterogeneous SIS spreading process, where both the infection rate per link and the recovery rate per node are heterogeneous. The infection rate of a link with weight *w*_*ij*_ is *β*_*ij*_ = *βw*_*ij*_. In network *G*_1_, which is unweighted, the infection rate is homogeneous. The heterogeneous recovery rate is motivated by the fact that airports with a larger declared capacity may recovery faster i.e. are more capable to deal with operational delay and congestion due to their better infrastructure. The declared capacity of an airport is affected by the number and geometric layout of the runways, type and location of taxiway exits from the runway and the ATM system. The primary factor in determining the capacity is the number of simultaneous active runways. The selection of runways to be operated depends on demand, weather conditions (visibility, wind speed/direction) and noise restrictions. During periods of high congestion, a large airport can decide to keep more runways active to match the demand, however, a small airport does not have that option. Furthermore, a large airport with several runways will have even more runway configurations, which is a combination of simultaneous active runways, weather conditions and assignment of aircraft types and movements (arrival/departures). This makes larger airports more suitable to handle congestion [29]. Similarly, recent studies showed that large airports are less likely to propagate delay [27, 28]. In the three networks we constructed, the node strength tends to be a good proxy of the declared capacity and it is strongly correlated with several other nodal centrality metrics. Hence, we define the recovery rate *δ*_*i*_ of a node as a function of its node strength:

$$\begin{array}{c} \delta_{i}=\delta (c+{\left(\frac{s_{i}}{s_{max}}\right)}^{\theta }) \end{array}$$ (1)

where node *i*’s strength is *s*_*i*_ = ∑_*j*_ *w*_*ij*_ and $s_{max}=\max_{1\leq i\leq N}\left\{s_{i}\right\}$. In the unweighted network topology *G*_1_, the strength *s*_*i*_ of a node *i* corresponds to its degree. The parameter *c* is a constant. The scaling factor *θ* ≥ 0 regulates to what extent the recovery rate of a node depends on the normalized node strength $\frac{s_{i}}{s_{max}}$. A large *c* results in a more homogeneous recovery rate, whereas a large *θ* leads to a high heterogeneity in recovery rate. When *θ* > 0 a node with a higher strength has a larger recovery rate. The heterogeneous SIS model coincides with the homogeneous one when *θ* = 0. The definition of the heterogeneous recovery rate Eq (1) is generic in the sense that it is a polynomial function of the node strength where the extent of homogeneity or heterogeneity can be tuned via parameter *c* and *θ*. The parameter set (*δ*, *c*, *θ*) will be calibrated or identified as the set that best reproduced the properties of the vulnerability of airports, as described in subsection Experiment description. The normalization by *s*_*max*_ in Eq (1) has no influence on the performance of the model but may ease the choice of the search space of *c* when we calibrate the parameters.

#### Individual-based mean-field approximation of the heterogeneous SIS model

We derive nodal infection probabilities via mean-field approximation instead of simulating the SIS stochastic process for computational efficiency. The N-Intertwined Mean-Field Approximation (NIMFA) is one of the most precise individual-based mean-field approximations [9]. Different from homogeneous or degree-based mean-field approximations where only the degree of a node is taken into account, NIMFA preserves the whole network topology in its governing equations, coupling the infection probability of neighboring nodes. It further assumes that the states of neighboring nodes are uncorrelated. Under NIMFA, the governing equation for a node *i* in our heterogeneous SIS spreading model is

$$\begin{array}{c} \frac{dv_{i}\left(t\right)}{dt}=-\delta_{i}v_{i}\left(t\right)+\left(1-v_{i}\left(t\right)\right)\sum_{j=1}^{N}\beta_{ij}v_{j}\left(t\right) \end{array}$$ (2)

where *v*_*i*_(*t*) is the infection probability of node *i* at time *t*, and *β*_*ij*_ = *βw*_*ij*_ is the infection rate associated to the link (*i*, *j*). In the meta-stable state, $\frac{dV(t)}{dt}=0$, where *V*(*t*) = [*v*_1_(*t*) *v*_2_(*t*) ⋯ *v*_*N*_(*t*)]^*T*^, lim_*t*→∞_ *v*_*i*_(*t*) = *v*_*i*∞_ and lim_*t*→∞_ *V*(*t*) = *V*_∞_. The infection probability of each node *V*_∞_ in the meta-stable state can be derived. The trivial all-zero solution corresponds to the absorbing state where all nodes are susceptible. The non-zero solution of *V*_∞_, if exists, indicates the existence of a meta-stable state with a non-zero fraction of infected nodes. Or else, the meta-stable state is 0 or not-existent. Given *θ*, *c* and the underlying network, the infection probability of each node remains the same if $\frac{\beta }{\delta }$ does not change. Without loosing the generality, we consider *β* = 1.

In a heterogeneous SIS model, the condition for the epidemic to spread out on a given network *G* is $\text{Re}(\lambda_{\text{1}}(\bar{A})>0$ where $\text{Re}(\lambda_{\text{1}}(\bar{A}))$ is the real part of the largest eigenvalue of the matrix $\bar{A}$, with its elements ${\bar{a}}_{ij}=\beta_{ij}$ if *i* ≠ *j* and ${\bar{a}}_{ij}=-\delta_{i}$ [36]. In particular, in our model *β*_*ij*_ = *w*_*ij*_, hence $\bar{A}=W-diag(\delta_{i})$. *δ*_*i*_ is defined according to Eq (1). Furthermore, the three network topologies *G*_1_, *G*_2_ and *G*_3_ are undirected: thus $\bar{A}$ is real and symmetric and $\lambda_{1}(\bar{A})$ is real. The condition $\text{Re}(\lambda_{1}(\bar{A}))>0$ becomes

$$\begin{array}{c} \lambda_{1}(\frac{1}{\delta }W-diag{\left(\frac{s_{i}}{s_{max}}\right)}^{\theta })>c \end{array}$$ (3)

### Evaluation methods

We evaluate our model via its capacity to capture: (a) the probability distribution of airport vulnerability and (b) the rank of airports in vulnerability.

#### Similarity of vulnerability and infection probability distribution

We firstly quantify the similarity of the probability distribution of nodal infection probability obtained from the heterogeneous SIS model with that of airport vulnerability via the Jensen Shannon divergence *JSD*. Given two discrete probability distributions *P* = (*p*_1_, *p*_2_, …, *p*_*K*_) and *Q* = (*q*_1_, *q*_2_, …, *q*_*K*_) where *K* ≥ 2, the Jensen-Shannon divergence(*JSD*) [37] measures the similarity of *P* and *Q*. We define the mixture of *P* and *Q* as *M* = (*m*_1_, *m*_2_, …, *m*_*K*_) where $m_{i}=\frac{p_{i}+q_{i}}{2}$, *i* ∈ {1, 2, …, *K*}. The Shannon’s entropy of of a distribution e.g. *P* is denoted as $H\left(P\right)=-\sum_{j=1}^{K}p_{j}\log_{2}p_{j}$. Jensen Shannon divergence measures the difference between the Shannon entropy of the mixture $M=\frac{1}{2}\left(P+Q\right)$ and the average Shannon entropy of P and Q, i.e.

$$\begin{array}{c} JSD\left(P,Q\right)=H\left(M\right)-\frac{1}{2}\left(H\left(P\right)+H\left(Q\right)\right) \end{array}$$ (4)

The Jensen-Shannon divergence is symmetric 0 ≤ *JSD*(*P*, *Q*) ≤ 1. A smaller JSD(*P*, *Q*) indicates a high similarity between the two distribution *P* and *Q*.

#### Airport ranking in vulnerability

From the application perspective, the identification of the most vulnerable airports is crucial. We can evaluate the quality of using nodal infection probability to rank airports in vulnerability as follows. A node with a high infection probability is supposed to correspond to an airport with a high vulnerability. We rank the nodes (airports) according to their infection probability and vulnerability respectively. These two rankings are recorded by two vectors $R^{v}=\left[R_{\left(1\right)}^{v},R_{\left(2\right)}^{v},\ldots ,R_{\left(N\right)}^{v}\right]$ and $R^{\phi }=\left[R_{\left(1\right)}^{\phi },R_{\left(2\right)}^{\phi },\ldots ,R_{\left(N\right)}^{\phi }\right]$ where $R_{\left(i\right)}^{v}$ is the index of the *i*-*th* highest node in infection probability and $R_{\left(i\right)}^{\phi }$ is the index of the *i*-*th* most vulnerable airport. The performance of using nodal infection probability to identify the top *f* fraction most vulnerable airports can be quantified by the top *f* recognition rate

$$\begin{array}{c} r_{\phi v}\left(f\right)=\frac{|R_{f}^{\phi }\cap R_{f}^{v}|}{|R_{f}^{\phi }|} \end{array}$$ (5)

where $R_{f}^{\phi }$ and $R_{f}^{V}$ are, respectively, the sets of nodes ranked in the top *f* fraction according to vulnerability and infection probability. $|R_{f}^{\phi }|=fN$ is the number of nodes in $R_{f}^{\phi }$. A higher recognition rate indicates a higher precision of using nodal infection probability to identify the top *f* fraction most vulnerable nodes.

We define the overall recognition quality *ξ* as the area under the *r*_*ϕv*_(*f*) function:

$$\begin{array}{c} \xi =\int_{0}^{1}r_{\phi v}\left(f\right)df \end{array}$$ (6)

The recognition quality 0 ≤ *ξ* ≤ 1 measures the overall performance of using infection probability to rank airports in vulnerability. The quality $\xi =\frac{1}{2}$ is obtained by the random ranking, which selects uniformly at random *f* fraction of nodes as the top *f* fraction most vulnerable ones. The maximum *ξ* corresponds to the case when *r*_*ϕv*_(*f*) = 1 ∀*f*, which means that *R*^*v*^ = *R*^*ϕ*^.

## Results and discussion

### Experiment description

Our heterogeneous SIS model has three control parameters *δ*, *c* and *θ*. In order to understand the influence of the parameters on the performance of the model, we consider all possible combinations of the parameters. We consider for *c* all possible values within [0, 2] and with step size 0.02. Similarly, *θ* can be any value within [0, 2] and with step size 0.1. The smaller step size of *c* is motivated by the high sensitivity of the model’s performances (especially the recognition quality *ξ*) on *c*. This is because the term ${\left(\frac{s_{i}}{s_{max}}\right)}^{\theta }$ in the recovery rate of a node can be small, when *θ* is large, especially in view of the heterogeneous node strength distribution (see Fig 2). Given the underlying network *G*_1_, *G*_2_ or *G*_3_, and given the parameter *c* and *θ*, the prevalence in the meta-stable state that can be derived via NIMFA is an increasing function of 1/*δ*. We consider the optimal value of *δ*, which is denoted as *δ*_*o*_, as the one that minimizes ${\left(E\left[\phi \right]-\frac{1}{N}\sum_{i=1}^{N}v_{i}\right)}^{2}$, i.e. when the average nodal infection probability is the closest to the average airport vulnerability. We obtained it via Brent optimization algorithm [38, 39]. For each possible *c*, *θ* and the underlying network *G*_1_, *G*_2_ or *G*_3_, which together determine the *δ*_*o*_, we derive the infection probability for each node via the NIMFA. The performance of the corresponding model is evaluated in comparison with the airport vulnerabilities via the Jansen-Shannon divergence *JSD* and the recognition quality *ξ*. We compare the performance of the heterogeneous SIS model on each network with the corresponding homogeneous model. In the baseline homogeneous SIS model on a given network, the infection rate of a link is *β*_*ij*_ = *w*_*ij*_, while the homogeneous recovery rate *δ*(*c* + 1) is tuned effectively as one parameter so that the average infection probability is the closest to the average vulnerability.

### Performance of the heterogeneous SIS model

**Fig 5:**
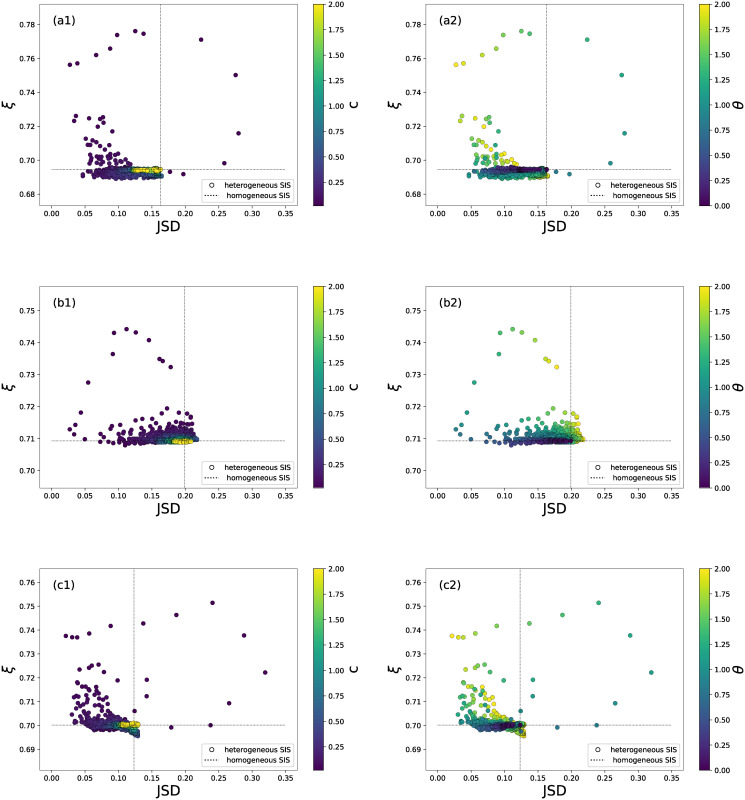
The scatter plot of the recognition quality *ξ* versus Jansen-Shannon divergence *JSD* for both heterogeneous and homogeneous SIS model with diverse parameter sets. The scatter plot is obtained in network *G*_1_ (figure a1, a2), *G*_2_ (b1, b2) and *G*_3_ (c1, c2). Points correspond to the heterogeneous model, where *θ* ∈ [0, 2] with step size 0.1 and *c* ∈ (0, 2] with step size 0.02. The points are colored according to parameter *c* in a1, b1, c1 and according to *θ* in a2, b2, c2. The dash lines correspond to the baseline homogeneous model.

The Jensen Shannon divergence *JSD* evaluates the similarity between nodal infection and vulnerability distribution, whereas the recognition quality *ξ* assesses the capability of identifying the most vulnerable airports according to their corresponding infection probabilities. In this section we explore the performance of the heterogeneous SIS model in comparison with the baseline homogeneous SIS model. If we aim to develop a model to reproduce the vulnerability distribution alone (to minimize the *JSD*) or the ranking of nodal vulnerability (to maximize *ξ*), but not both at the same time, the heterogeneous SIS model evidently outperforms the homogeneous one. As shown in Fig 5, the minimal possible *JSD* and the maximal *ξ* achieved by the heterogeneous model are far lower and higher respectively than those obtained by the homogeneous model. The minimal *JSD* and the maximal *ξ* are not obtained by the heterogeneous model at the same time, i.e. via the same parameter set *θ* and *c*.

Furthermore, the data points on the top-left panel in each sub-figure of Fig 5 correspond to the parameter sets with which the heterogeneous model outperforms the homogeneous one in reproducing both the vulnerability distribution and ranking the airports in vulnerability. Among those points, those that lead to an evidently high recognition quality are within the parameter range *θ* > 1 and *c* = 0.02, when the recovery rate is highly heterogeneous. The heterogeneous SIS model on the unweighted network *G*_1_ could possibly achieve slightly better recognition quality than the model on *G*_2_ and *G*_3_. The homogeneous model on network *G*_1_ however, performs worse than that on *G*_2_ and *G*_3_ in recognition quality. The network *G*_1_, which contains less information than the other two networks, is sufficient for the heterogeneous model to perform well. When *c* = 0.02, the heterogeneous model achieves the best performance in *ξ*. This suggests that a fine tuning of the *c* within the range (0, 0.02) may further improve the performance of the model. The parameter sets that we have considered are sufficient for us to illustrate that the heterogeneous SIS model could perform better than the homogeneous one.

### The infection probability versus the node strength of a node

Identifying the most vulnerable airports is crucial for operations. In this section, we aim to understand why the heterogeneous SIS model better recognizes vulnerable airports, i.e. is higher in recognition quality than the homogeneous model. In the homogeneous SIS model, a node with a large strength tends to have a high infection probability. In the heterogeneous SIS model, a node with a large strength has high rates of getting infected by its neighbors, contributing to a high infection probability. On the other hand, a node with a large strength could have a large recovery rate when *θ* > 0. These two factors imply that a node with a large node strength does not necessarily have a high infection probability. In this section, we explore whether the better performance of our heterogeneous SIS model in recognition quality corresponds to its better capability to reproducing the relationship between the vulnerability and strength of a node if compared to the homogeneous SIS model.

**Fig 6:**
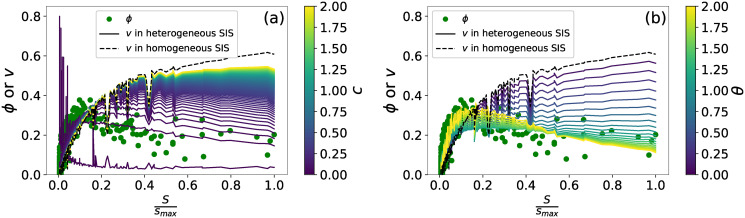
Airport vulnerability and nodal infection probability versus normalized node strength. The scatter plot of the vulnerability *ϕ* (points) and infection probability *v* (lines) of a node versus the normalized node strength $\frac{s_{i}}{s_{max}}$ of the node on the underlying network topology *G*_1_. The black dashed line corresponds to the baseline homogeneous model (*θ* = 0). Solid lines correspond to the heterogeneous model with *θ* = 1.50, colored according to the parameter *c* (figure a) or with *c* = 0.02, colored according to the parameter *θ* (figure b).

We take network *G*_1_ as an example. The heterogeneous SIS model on *G*_1_ achieves the highest recognition quality *ξ* when *c* = 0.02 and *θ* = 1.5. We consider the SIS model when *c* = 0.02 whereas *θ* varies and when *θ* = 1.5 whereas *c* varies. We plot the vulnerability *ϕ* and the meta-stable infection probability *v* (derived by the heterogeneous SIS model or the homogeneous SIS baseline model) of a node versus the strength of the node in Fig 6a. When *θ* < 1, and *c* = 0.02, the infection probability increases monotonically with the strength of a node (see Fig 6a). When *θ* > 1, the new phenomena unfolds: high nodal infection probability is obtained by nodes with an intermediate strength, but not those having a small nor large strength. A large *θ* attributes to the heterogeneity of the recovery rates, allowing nodes with a large strength to have a small infection probability. Given the *θ* = 1.5, Fig 6b shows that the nodal infection probability increases monotonically with the node strength when *c* is large, e.g. *c* > 1. A large *c* reduces the heterogeneity of the recovery rate. When *c* is small, the maximal vulnerability has also been obtained by nodes with an intermediate node strength.

**Fig 7:**
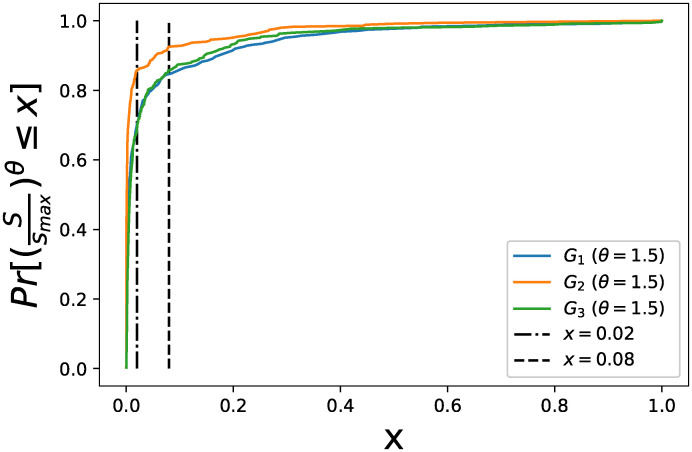
Cumulative distribution of ${\left(\frac{S}{s_{max}}\right)}^{\theta }$ in each network when *θ* = 1.5.

The node strength that leads to the maximal infection probability increases as *c* increases because a larger *c* makes the recovery rate more homogeneous. In the extreme case, the most heterogeneous case, when *θ* > 1 and *c* = 0, *v* decreases monotonically as the node strength increases, which can be seen in Fig 6a. In this special case, a larger *θ* > 1 corresponds to a steeper decrease. The relative magnitude of the constant term *c* with respect to the node strength dependant term ${\left(\frac{s_{i}}{s_{max}}\right)}^{\theta }$ of *δ*_*i*_ decides when the phenomena occurs that the infection probability increases first and decreases afterward as the node strength increases. Fig 7 illustrates the cumulative distribution $Pr[{\left(\frac{S}{s_{max}}\right)}^{\theta }\leq x]$ of the term ${\left(\frac{s_{i}}{s_{max}}\right)}^{\theta }$. The model on *G*_1_ that maximizes the recognition quality is obtained when *θ* = 1.5 and *c* = 0.02 (observed within the range we have searched for). In this case, the constant *c* is larger than the term ${\left(\frac{s_{i}}{s_{max}}\right)}^{\theta }$ of *δ*_*i*_ in less than 70% of the nodes. The model where ${\left(\frac{s_{i}}{s_{max}}\right)}^{\theta }\leq c$ in most nodes (e.g. when *c* = 0.1 and *θ* = 1.5) is not optimal. These observations motivate that we may identify the optimal parameter set more efficiently by better choosing the search space.

## Conclusion

We model airport traffic congestion contagion as a heterogeneous SIS spreading process on an airport transportation network, aiming to identify airport’s vulnerability, i.e. probability of being congested, using nodal infection probabilities derived from our model. Three airline networks are constructed to capture diverse information e.g. flight frequency and duration and the infection rate of each link is assumed to be proportional to its link weight. Per node, we introduce an heterogeneous recovery rate which is a function of its node strength. The model is evaluated via its capability to reproduce the distribution of nodal vulnerability and to rank airports in vulnerability. Our model evidently outperforms the SIS model with a homogeneous recovery rate in ranking airports from both perspectives. One explanation of the better performance of our heterogeneous model in reproducing the ranking of airports in vulnerability is that: the phenomena that the vulnerability is the largest at airports whose strength in the airline network is neither too large nor too small can be only captured by the heterogeneous model. In particular, a node with a large strength has high rates (link weights) of getting infected by its neighbors, whereas its large recovery rate could reduce its infection probability. Finally, the simplest airline network that represents which airports have direct flight(s) in between already allows the heterogeneous model to evidently outperform the homogeneous one.

The identification of vulnerable airports is crucial for airport operations. Beyond, our model may facilitate the development and evaluation of optimization strategies. The optimization problem can be, e.g. which airports should be invested in improving their capacity thus reducing their vulnerability or in improving their recovery rates in order to minimize the global vulnerability. The derived model that describes how congestion at one airport spreads to other airports could be used to evaluate optimization solutions as a starting point. Such questions require as well further improvement and validation of the model, accounting for e.g. other operational factors and the time varying nature of airport vulnerability. The definition of airport vulnerability can also be generalized by considering e.g. the extent of congestion at an airport.

## References

[1] Albert R, Barabási AL. Statistical mechanics of complex networks. Reviews of modern physics. 2002;74(1):47 10.1103/RevModPhys.74.47

[2] Newman ME. The structure and function of complex networks. SIAM review. 2003;45(2):167–256. 10.1137/S003614450342480

[3] Boccaletti S, Latora V, Moreno Y, Chavez M, Hwang DU. Complex networks: Structure and dynamics. Physics Reports. 2006;424(4-5):175–308. 10.1016/j.physrep.2005.10.009

[4] Kiss IZ, Miller JC, Simon PL, et al Mathematics of epidemics on networks. Cham: Springer 2017;598 10.1007/978-3-319-50806-1

[5] Zanin M, Lillo F. Modelling the air transport with complex networks: A short review. Eur Phys J Spec Top. 2013;215(1):5–21. 10.1140/epjst/e2013-01711-9

[6] Pastor-Satorras R, Castellano C, Van Mieghem P, Vespignani A. Epidemic processes in complex networks. Rev Mod Phys. 2015;87(3):925 10.1103/RevModPhys.87.925

[7] Barrat A, Barthelemy M, Vespignani A. Dynamical processes on complex networks. Cambridge University Press; 2008 10.1017/CBO9780511791383

[8] Li D, Qin P, Wang H, Liu C, Jiang Y. Epidemics on interconnected lattices. EPL (Europhysics Letters). 2014;105(6):68004 10.1209/0295-5075/105/68004

[9] Van Mieghem P, Omic J, Kooij R. Virus spread in networks. IEEE/ACM Transactions On Networking. 2008;17(1):1–14. 10.1109/TNET.2008.925623

[10] Qu B, Wang H. SIS epidemic spreading with correlated heterogeneous infection rates. J Phys A. 2017;472:13–24. 10.1016/j.physa.2016.12.077

[11] Qu B, Wang H. SIS epidemic spreading with heterogeneous infection rates. IEEE TNSE. 2017;4(3):177–186. 10.1109/TNSE.2017.2709786

[12] Li C, van de Bovenkamp R, Van Mieghem P. Susceptible-infected-susceptible model: A comparison of N-intertwined and heterogeneous mean-field approximations. Phys Rev E. 2012;86(2):026116 10.1103/PhysRevE.86.026116 23005834

[13] Van Mieghem P. The N-intertwined SIS epidemic network model. Computing. 2011;93(2-4):147–169. 10.1007/s00607-011-0155-y

[14] Li C, Wang H, Van Mieghem P. Epidemic threshold in directed networks. Phys Rev E. 2013;88(6):062802 10.1103/PhysRevE.88.062802 24483506

[15] Yang Z, Zhou T. Epidemic spreading in weighted networks: An edge-based mean-field solution. Phys Rev E. 2012;85(5):056106 10.1103/PhysRevE.85.056106 23004820

[16] Lu D, Yang S, Zhang J, Wang H, Li D. Resilience of epidemics for SIS model on networks. Chaos Interdiscip J Nonlinear Sci. 2017;27(8):083105 10.1063/1.4997177 28863477

[17] Qu B, Li C, Van Mieghem P, Wang H. Ranking of nodal infection probability in susceptible-infected-susceptible epidemic. Scientific Reports. 2017;7(1):1–10. 10.1038/s41598-017-08611-9 28835611 PMC5569095

[18] Barrat A, Barthelemy M, Pastor-Satorras R, Vespignani A. The architecture of complex weighted networks. Proceedings of the National Academy of Sciences. 2004;101(11):3747–3752. 10.1073/pnas.0400087101 PMC374315 15007165

[19] Guimerà R, Mossa S, Turtschi A, Amaral LN. The worldwide air transportation network: Anomalous centrality, community structure, and cities’ global roles. Proceedings of the National Academy of Sciences. 2005;102(22):7794–7799. 10.1073/pnas.0407994102 PMC1142352 15911778

[20] Reggiani A, Signoretti S, Nijkamp P, Cento A. Network measures in civil air transport: a case study of Lufthansa In: Networks, Topology and Dynamics. Springer; 2009 p. 257–282. 10.1007/978-3-540-68409-1_14

[21] Han DD, Qian JH, Liu JG. Network topology and correlation features affiliated with European airline companies. Phys A. 2009;388(1):71–81. 10.1016/j.physa.2008.09.021

[22] Chi L, Cai X. Structural changes caused by error and attack tolerance in US airport network. Int J Mod Phys B. 2004;18(17n19):2394–2400. 10.1142/S0217979204025427

[23] Wilkinson SM, Dunn S, Ma S. The vulnerability of the European air traffic network to spatial hazards. Natural Hazards. 2012;60(3):1027–1036. 10.1007/s11069-011-9885-6

[24] Fleurquin P, Ramasco JJ, Eguiluz VM. Systemic delay propagation in the US airport network. Scientific Reports. 2013;3:1159 10.1038/srep01159 23362459 PMC3557445

[25] Ciruelos C, Arranz A, Etxebarria I, Peces S, Campanelli B, Fleurquin P, et al. Modelling delay propagation trees for scheduled flights. In: Proceedings of the 11th USA/EUROPE Air Traffic Management R&D Seminar, Lisbon, Portugal; 2015. p. 23–26.

[26] Baspinar B, Koyuncu E. A data-driven air transportation delay propagation model using epidemic process models. International Journal of Aerospace Engineering. 2016;2016 10.1155/2016/4836260

[27] Belkoura S, Zanin M. Phase changes in delay propagation networks. arXiv preprint arXiv:161100639 [physics.soc-ph]. 2016;.

[28] Zanin M, Belkoura S, Zhu Y. Network analysis of chinese air transport delay propagation. Chinese Journal of Aeronautics. 2017;30(2):491–499. 10.1016/j.cja.2017.01.012

[29] De Neufville R, Odoni A. Airport Systems. Planning, Design and Management; 2003.

[30] Lacasa L, Cea M, Zanin M. Jamming transition in air transportation networks. J Phys A. 2009;388(18):3948–3954. 10.1016/j.physa.2009.06.005

[31] Saberi M, Hamedmoghadam H, Ashfaq M, Hosseini SA, Gu Z, Shafiei S, et al A simple contagion process describes spreading of traffic jams in urban networks. Nature Communications. 2020;11(1):1–9. 10.1038/s41467-020-15353-2 32265446 PMC7138808

[32] United States Bureau of Transportation Statistics.;. http://www.transtats.bts.gov.

[33] Köstler K, Gobardhan R, Ceria A, Wang H. Modeling Airport Congestion Contagion by SIS Epidemic Spreading on Airline Networks. In: International Conference on Complex Networks and Their Applications. Springer; 2019. p. 385–398. 10.1007/978-3-030-36687-2_32

[34] Onnela JP, Saramäki J, Kertész J, Kaski K. Intensity and coherence of motifs in weighted complex networks. Phys Rev E. 2005;71(6):065103 10.1103/PhysRevE.71.065103 16089800

[35] Newman ME. Scientific collaboration networks. II. Shortest paths, weighted networks, and centrality. Phys Rev E. 2001;64(1):016132 10.1103/PhysRevE.64.016132 11461356

[36] Ottaviano S, De Pellegrini F, Bonaccorsi S, Mugnolo D, Van Mieghem P. Community Networks with Equitable Partitions In: Multilevel Strategic Interaction Game Models for Complex Networks. Springer; 2019 p. 111–129. 10.1007/978-3-030-24455-2_6d

[37] Lin J. Divergence measures based on the Shannon entropy. IEEE Transactions on Information theory. 1991;37(1):145–151. 10.1109/18.61115

[38] Brent RP. An algorithm with guaranteed convergence for finding a zero of a function. The Computer Journal. 1971;14(4):422–425. 10.1093/comjnl/14.4.422

[39] Press WH, Teukolsky SA, Flannery BP, Vetterling WT. Numerical recipes in Fortran 77 vol.1: the art of scientific computing. Cambridge university press; 1992.

